# Supplementary material for: A method for non-destructive microwave focusing for deep brain and tissue stimulation
Source: PLoS One. 2023 Feb 9;18(2):e0278765. doi: 10.1371/journal.pone.0278765 (PMC9910646; doi:10.1371/journal.pone.0278765)
Supplement: Attachment — Submitted filename: Review Responses - PLOS One.pdf [file pone.0278765.s001.pdf]

Reviewer #1: In this manuscript, Harid and colleagues developed a theoretical framework to flexibly use an array of microwave antennas to selectively stimulate body parts with higher spatial resolution than other techniques using lower frequency stimulation. This study is timely, since there is an increased interest in non-invasive stimulation techniques/approaches that can achieve higher spatial resolution.

While I think the manuscript has the potential to be appealing for a large audience, major improvements are required.

Major comments

A - I think the authors should provide more technical details. In particular, the absence of a methods section is unusual. For example, while I do not think that a full derivation of the equations used in this manuscript is necessary, it would help the reader to get a quantitative sense of how they can be derived. Furthermore, some details are actually completely missing. For example, unless I missed that, I did not find how the numerical estimation of the Green functions is computed (also, was any specific software used for that?). Thus, I think that in general this manuscript will benefit from having more technical details in a methods section.

We thank the reviewer for the useful feedback. We have updated the name of the subsection titled "Microwave Fields from Multiple Transmitters" to "Method for Calculating Microwave Fields from Multiple Transmitters". We have re-written **Lines 129-168** to provide a more detailed description on how the Green's functions are calculated and used before being utilized in the optimization algorithm.

We have also updated the name of the section titled "MW Focusing Using an Optimized 3D Antenna Array" to "Method for MW Focusing Using an Optimized 3D Antenna Array". To improve clarity in this section, we have added **lines 189-192** that reads, "There are many potential methods that can be employed to determine the optimal amplitudes of each of the magnetic dipoles. The most common approach is to formally define an "error" between the model and desired field profile, then numerically determine the magnetic dipole amplitudes that minimize the error. For this study, …"

B - The electric fields that are reported in the main figures are extremely weak. A peak amplitude of 0.015 V/m represents a weak field, even compared to the ones induced by transcranial direct current stimulation (tDCS) or transcranial alternating current stimulation (tACS), which are in the order of max 1 V/m (already ~100 times lower than the ones induced in the brain during TMS). In the field of transcranial stimulation there is an ongoing discussion on whether fields of this amplitude are strong enough to induce any effect on neuronal activity at all. Can the intensity of the stimulation reported in this study be increased to achieve higher amplitude electric fields? What are the safety limitations (max intensity that can be used without inducing tissue damage, likely due to heating)? These questions are quite critical to establish the feasibility of MW stimulation in real-case scenarios.

We thank the reviewer for making this point, it is indeed an important consideration to explore further in the text. The physical description of microwave penetration into tissue is assumed to be linear for this problem. This of course assumes that heating effects are negligible. Prior work by *Seo et al.,2018* suggests changes to firing rates are statistically measurable for radiated power as low as 4 mW. In addition, according to FCC electromagnetic safety guidelines, the maximum permissible exposure limits for electric and magnetic fields are hundreds of V/m and several A/m at high frequencies. Therefore, at these low excitation values, local heating should not be an important factor of tissue damage. Thus, given the linearity of the system, we set the

peak value of the desired electric field to be equal to unity. If the peak value needs to be higher, all the antenna amplitudes can be scaled proportionately to obtain the new peak value.

We have added **Lines 224-228** in the manuscript to make it more clear to the reader:

"It is worth mentioning that in all cases, the peak desired electric field is set to 1 V/m. As described in the previous section, the mathematical description of microwave penetration into tissue is assumed to be linear (i.e heating effects are negligible). As such, if the peak value needs to be higher in an application, all the antenna amplitudes can simply be scaled proportionately to obtain the new peak value".

It is also worth noting that, apart from a handful of studies, not much work has been done to look at the threshold microwave power levels required to induce changes in neuron firing rates. As such, this is very much still an open area of research and is the reason why our manuscript is focused first on building a theoretical methodology for microwave-based stimulation. We are actively running patch-clamp experiments with microwave stimulation in our lab, this will be the focus of follow-up work on the subject.

C - I think the discussion should be improved. For instance, what are the benefits of MW stimulation compared, for example, to transcranial ultrasound stimulation (for example Folloni et al., Neuron 2019) or temporal interference (TI) stimulation (for example, Grossman et al., Cell 2017)? It seems to me that all these techniques try to achieve higher spatial resolutions, so a direct comparison would enrich this manuscript. In general a discussion about the tradeoff between focality/intensity/safety (related to point B) is quite necessary in this manuscript.

We thank the reviewer for pointing out these two local stimulation techniques. These three different techniques are indeed important for future developments of non-invasive local neural modulation. These different approaches are not mutually exclusive and can be complementary to different clinical and other applications. We have added a paragraph in the discussion to point out and to compare these different techniques.

D - Since a Methods section is missing, so is the "Data Availability Statement" which should describe where the data/code used/generated in this study will be found.

We thank the reviewer for the suggestion, we have added a Data Availability section at the end of the manuscript.

Minor comments

1 - Line 24: "For instance, Deep Brain Stimulation (DBS) requires incision of an extended electrode deep into…".

Incision -> insertion?

This has been corrected in the updated manuscript.

2 - Line 60: "relies on the surgical incision of a MW antenna".

Incision -> insertion?

This has been corrected in the updated manuscript.

3 - Line 63: "For instance, (20) showed that modulated MW signals can also be used to vary firing rates of neurons in mice".

I think reference 20 is not the correct one.

Yes, this was incorrect and we thank the reviewer for noting this. It has been changed to (7) instead in the updated manuscript. The citation corresponds to the following paper:

Seo T, Oh S, Jung D, Huh Y, Cho J, Kwon Y. Noninvasive Brain Stimulation Using a Modulated Microwave Signal. Journal of electromagnetic engineering and science. 2018;18(1):70–2.

4 - Line 70: "However, three-dimensional MW focusing within biological tissue has not been adequately investigated in the past due to the complexity of modeling MW signal propagation through the human body". The authors should mention exactly why it is complex to model MW signal propagation through the human body.

We have updated the manuscript to include the following sentence on **lines 72-73**. "MW signals, once inside the human body, will have wavelengths that are shorter than in free space and can exhibit complicated scattering and diffraction".

5 - Line 95: "by the electrical conductivity , dielectric permittivity , conductivity σ,"

Conductivity is mentioned twice.

The sentence has been corrected on **Lines 97-98.**

6 - Line 100: equation 1.

Is \mu the magnetic permeability? If so, it is not defined in the text.

Yes, it is the permeability of free space. The manuscript has been updated on **Line 103** to say "Here, \mu is the permeability of free space."

7 - Line 139: "numerical Green's functions using FEKO,"

"FEKO" is not defined.

FEKO is an electromagnetic simulation package that was utilized for this work. We have updated the manuscript in **Lines 145-148** to say:

"The Green's functions are computed using the commercial electromagnetic simulation package, FEKO. FEKO utilizes a hybrid finite element and boundary element methodology to compute electric and magnetic fields in arbitrary material structures due to arbitrary sources."

8 - Line 297: "appropriately driving a surrounding the region by an array of antennas"

Something is missing in that sentence. I think the authors meant "appropriately driving an array of antennas surrounding the region".

We thank the reviewer for pointing out this mistake. The updated manuscript has been corrected on **Line 322** to say:

“..inside a target of interest by appropriately driving an array of antennas that surround the region“

9 - Line 326: “For instance, surgeries of deep brain stimulation are currently only reserved for on patients who have developed advanced drug-resistive symptoms due to high invasiveness.” This sentence is not very clear (and there are some typos). I think that the “invasiveness” (I guess of the electrodes implantation) is not what causes the development of “drug-resistive symptoms”.

The updated manuscript has been corrected on **lines 352-353** to say:

“For instance, surgeries of deep brain stimulation are currently only reserved for patients who have developed advanced drug-resistive symptoms since the procedure is otherwise considered excessively invasive”

10 - Caption Fig.8: “The material is a brain”

I would not call the brain a “material”. I think the authors meant “gray matter” (if the parameters used correspond to gray matter properties)?

The updated manuscript has been corrected on the figure caption to say:

“The material properties are consistent with gray matter…”

Reviewer #2: A proper review cannot be done, the results are so poorly displayed that I cant **evaluate the results.**

Figures should be improved.

A proper review cannot be done, the results are so poorly displayed that I cant evaluate the results.

Figures should be improved.

We apologize for the issue. It seems that the figure(s) resolution was severely affected on the submission site. Hopefully this will not be the case during resubmission.
