# Supplementary material for: A method for non-destructive microwave focusing for deep brain and tissue stimulation
Source: PLoS One. 2023 Feb 9;18(2):e0278765. doi: 10.1371/journal.pone.0278765 (PMC9910646; doi:10.1371/journal.pone.0278765)
Supplement: Attachment — Submitted filename: Response_to_Reviewers_PLOS_ONE2.pdf [file pone.0278765.s002.pdf]

Response to Review Comments (PLOS ONE)

Please use the space provided to explain your answers to the questions above. You may also include additional comments for the author, including concerns about dual publication, research ethics, or publication ethics. (Please upload your review as an attachment if it exceeds 20,000 characters)

Reviewer #1: In this revision Harid and colleagues properly addressed all my previous comments. I now understand the methods much better and I am therefore quite pleased with this revised version of the manuscript.

Thank you very much for addressing all my comments.

Just a small detail:

Line 146: "to be calculated using an electromagnetic simulation code."

I would write software/program instead of "code".

We have changed the line to:

"...electromagnetic simulation program"

Reviewer #3: The authors addressed the remarks from reviewer #1. For reviewer #2, the figures are still not well visible in the core of the article but I could download them one by one separately. However, since the background is transparent some axis labels are not visible (especially figs 6, 7, 8 ... h, g, l; d, i, n and; e, j, o). It would require having the figure separately with white background.

As a brief review, the article is of interesting content for exploring the feasibility of creating MW focal spots inside the human body for either neuro-stimulation or potentially non invasive MW thermal ablation. A few minor revisions need to be adressed:

fig 6 / 7 / 8 : what does 15n / 10n / 5n mean ?

We have fixed this in the revised manuscript. The dipole amplitudes are in units of 'nA-m^2' (nano-Amp-meter-squared), which is now shown on figures 6/7/8.

The goal is to achieve 1V / m at the focal spot however values are much below in the simulation (c, h, m and d, i, n). How do you explain this?

We thank the reviewer for pointing this out, it is an error on our part. The desired field was set 0.01 V/m at the focal spot. This has been corrected on **line 226.**

> as said before, the figures are not easily visible since the background is transparent, it is hard to see the labels when downloading them one by one

The figure(s) quality has been corrected and reuploaded.

> what are the phase shifts necessary to apply for each individual antenna to obtain constructive interference at target ?

The phase shift between neighboring antennas (for a given polarization) is on the order of 0.2-2 radians with larger phase shifts for the simulations with fewer antennas. The exact phase values emerge as a result of the optimization from equation (6). It is also worth noting that the magnetic dipole antennas have three independent phases associated with the three directions of polarization. This information has been added to the revised manuscript on **lines 262-264.**

> some hardware consideration would be very interesting to discuss. Being able to control the power and the phase shifts of 64 individual antennas at 1GHz seems to be a challenging problem.

We appreciate the suggestion from the reviewer. In accordance, we have added the following statement to **lines 337-342**:

"In the current framework, each transmitting antenna is assumed to have a magnitude and phase that is independently controllable. Practically, driving each antenna with its own input can be a difficult engineering task. However, in the past several years, advances in antenna array technology makes the possibility of controlling hundreds of antennas quite viable. For instance, active electronically steered arrays (AESA) are routinely used in modern radar and MIMO systems. As such, aside cost limitations, the optimization methodology described herein is reasonable to implement with existing array technology."

> L.377: "the technique requires good probe contact": would not it be the same with the antennas ?" at 1GHz, would the electric field be able to propagate without good contact ? I may be wrong but in thermal ablation, bad tissue contact means high reflected power and low transmission due to permittivity mismatch between air and tissue.

The reviewer brings up a good point, and the explanation is somewhat nuanced.The primary difference is due to the frequency of operation. The method described in *Grossman et al., 2017* utilizes electric fields in the few kilohertz range. In this frequency band, emitting electric fields through the air is inefficient, and thus requires direct electrode contact with the scalp to directly drive currents through conduction. Essentially the patient's head becomes part of a low frequency circuit. At 1GHz however, the antennas can emit RF fields much more efficiently and do not necessarily need direct probe contact. Furthermore, the impedance (or permittivity) mismatch can be somewhat overcome by the near-field characteristics of loop antennas (see **Harid, Vijay, et al. "Magnetic field penetration into a metal enclosure using an ELF/VLF loop antenna."** ***IEEE Transactions on Electromagnetic Compatibility*** **62.4 (2019): 1225-1236.**).

We have added some of this information in the updated manuscript on **lines 135-136.**
